# Telling the national trachoma elimination story through the dossier

**Published:** 2025-01-31

**Figure:**
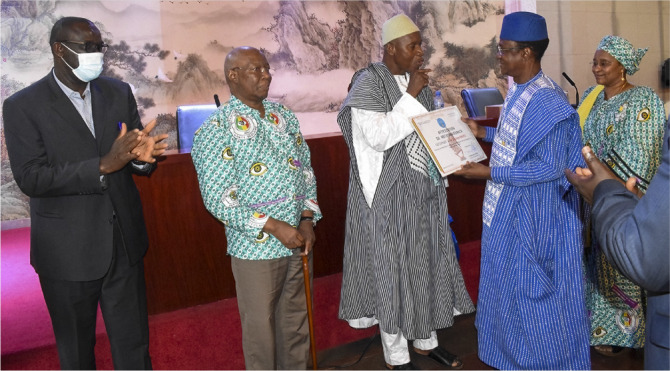
Mali received official recognition from the World Health Organization (WHO) for eliminating trachoma as a public health problem on May 16, 2023.

© MINISTRY OF HEALTH, MALI CC BY-NC-SA 4.0

Trachoma is the leading infectious cause of blindness worldwide. The fight against trachoma has been highlighted in the 2019 World Report on Vision as one of the many successes in eye care over the last few decades.^1^ As of 21 October 2024, 21 countries have successfully submitted trachoma elimination dossiers, resulting in validation by the World Health Organization (WHO) of the elimination of trachoma as a public health problem in these countries.^2^ The dossiers have a dual role: to provide evidence of trachoma elimination and to document how elimination will be maintained. Sustaining elimination requires continuous improvements in hygiene and the environment to prevent a recrudescence of trachoma, and the integration of trachomatous trichiasis (TT) surgery into routine eye health services.^3^

Strengthening the capacity of national trachoma programme staff members to prepare dossiers, in collaboration with relevant national stakeholders, is important to ensure national buy-in and long-term commitment for surveillance, WASH activities, and the management of incident TT cases.

WHO, non-governmental organisations (NGOs) and the WHO Collaborating Centre for Trachoma at the University of Cape Town, South Africa, have supported three regional workshops (in Francophone Africa, Anglophone Africa, and the Eastern Mediterranean Region) on trachoma elimination dossiers.

Some of the key messages and lessons learned from these workshops and from the development of trachoma elimination dossiers include:

- It is never too early to begin the process of writing the dossier. Several descriptive and historical sections can be written while interventions are underway. The Microsoft Excel data sheet and narrative sections on the SAFE strategy can be drafted and updated regularly as new data become available. Other health sectors and ministries may be needed to provide background information and data. Early involvement of these entities can ensure the timely sharing of data.
- WHO provides templates for developing the dossier and there have been minor adjustments to the templates for the narrative section and the Excel data sheet, most recently in August 2023. Countries that are already well advanced in their dossiers do not need to switch to the adjusted templates. The templates provide useful suggestions for organising information in the dossier, but national programmes can organise their dossier as they see fit, as long as they clearly demonstrate that they have met the three criteria for validation.^4^
- Many countries have established a local dossier working group, while others have used a consultant (international or in-country). It is important for health ministries to “own” the dossier development process. National programmes should consider having one person who writes well as the lead writer to ensure consistency in style and to help readability.
- Writing the dossier involves centralising and synthesising a large amount of qualitative and quantitative data. Interviews with former programme staff may be conducted to capture key milestones, achievements, and decision-making in the implementation of the trachoma programme.
- Effective data management is essential for minimising time searching for information, enhancing productivity, and ensuring the accuracy and security of data. Centralised storage of all publications referenced in the dossier (Global Trachoma Mapping Project and Tropical Data reports, programme report files, and other documents) may help. Records that have not been published in peer-reviewed journals should be stored securely (in a cloud-based server, for example) to permanently protect the information and to have it available in case the Dossier Review Group asks to see it.
- The length of the dossier is context-specific but it should be concise, providing the necessary evidence that elimination has been achieved.
- Some topics, such as approaches to document full geographic coverage of TT, justifying why some evaluation units were not mapped, the use of alternative indicators, and strategies for post-validation surveillance, required in-depth discussions at the workshops. Guidance for programme implementation and dossier presentation in relation to these activities is mostly country-specific.
- Making dossiers available after validation assists other countries as they develop their own dossiers.

Trachoma elimination dossiers offer a unique opportunity for countries to showcase their success. While countries must lead the development process, various approaches can support health ministries in continuing their pathway to successful dossier submission. WHO, WHO Collaborating Centres for Trachoma, and NGO partners can be contacted for tailored support if that support is not already in place. Other useful documents on dossier development have been synthesised previously.^5,6^ For further information on trachoma dossiers, please contact Dr Amir Kello at kelloa@who.int.
